# Inhibition of VDAC1 oligomerization blocks cysteine deprivation-induced ferroptosis via mitochondrial ROS suppression

**DOI:** 10.1038/s41419-024-07216-1

**Published:** 2024-11-09

**Authors:** Se-Kyeong Jang, Se Hee Ahn, Gyeongmi Kim, Selim Kim, Jungil Hong, Ki Soo Park, In-Chul Park, Hyeon-Ok Jin

**Affiliations:** 1 Division of Fusion Radiology Research, Korea Institute of Radiological & Medical Sciences, Seoul, Republic of Korea; 2 Department of Food Science and Technology, College of Science and Convergence Technology, Seoul Women’s University, Seoul, Republic of Korea; 3 Department of Biological Engineering, Konkuk University, Seoul, Republic of Korea; 4 KIRAMS Radiation Biobank, Korea Institute of Radiological and Medical Sciences, Seoul, Republic of Korea

**Keywords:** Cancer microenvironment, Cancer metabolism

## Abstract

Ferroptosis, a regulated form of cell death dependent on reactive oxygen species (ROS), is characterized by iron accumulation and lethal lipid peroxidation. Mitochondria serve as the primary source of ROS and thus play a crucial role in ferroptosis initiation and execution. This study highlights the role of mitochondrial ROS and the significance of voltage-dependent anion channel 1 (VDAC1) oligomerization in ferroptosis induced by cysteine deprivation or ferroptosis-inducer RSL3. Our results demonstrate that the mitochondria-targeted antioxidants MitoQ and MitoT effectively block ferroptosis induction and that dysfunction of complex III of the mitochondrial electron transport chain contributes to ferroptosis induction. Pharmacological inhibitors that target VDAC1 oligomerization have emerged as potent suppressors of ferroptosis that reduce mitochondrial ROS production. These findings underscore the critical involvement of mitochondrial ROS production via complex III of the electron transport chain and the essential role of VDAC1 oligomerization in ferroptosis induced by cysteine deprivation or RSL3. This study deepens our understanding of the intricate molecular networks governing ferroptosis and provides insights into the development of novel therapeutic strategies targeting dysregulated cell death pathways.

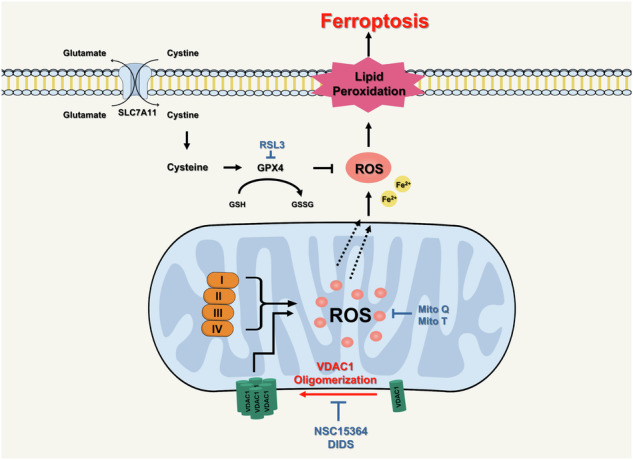

## Introduction

Ferroptosis is a form of regulated cell death (RCD), characterized by the accumulation of lipid peroxidation products and lethal reactive oxygen species (ROS) derived from iron metabolism [1, 2]. It is triggered by inhibiting the glutamate/cystine antiporter, activating iron transporters, or inhibiting glutathione peroxidase 4, an important intracellular antioxidant enzyme at the cellular level [2, 3]. Cysteine plays a crucial role in preventing ferroptosis by influencing the synthesis of essential molecules such as glutathione, coenzyme A, and iron-sulfur clusters [4]. These molecules are vital for maintaining the antioxidant network that protects against ferroptosis and for regulating intracellular iron distribution, a critical catalyst in this process [4]. Consequently, cysteine deprivation under conditions such as nutrient limitation or experimental cysteine depletion disrupts antioxidant systems and sulfur redox homeostasis, which can impair cellular function and contribute to aging and various diseases, such as cancer, cardiovascular disease, and neurodegenerative disease [5].

Ferroptosis presents unique morphological features that distinguish it from other RCD pathways, including apoptosis, necroptosis, and pyroptosis [1, 6]. Notably, ferroptotic cells are characterized by smaller mitochondria with condensed mitochondrial membrane densities, reduced or disappeared mitochondrial cristae, and rupture of the outer mitochondrial membrane (OMM) [2, 7]. The alterations in mitochondrial morphology observed during ferroptotic processes accentuate the relationship between ferroptosis and mitochondrial function [2]. Recently, ferroptosis has gained considerable attention in cancer research because of its pivotal role in tumor suppression and its potential as an Achilles heel in therapy-resistant cancers [8, 9]. Targeted modulation of ferroptosis presents a promising avenue for enhancing the effectiveness of immunotherapy and overcoming resistance to established cancer treatments [10, 11]. Additionally, preclinical studies have revealed synergistic effects and favorable tolerability with the use of ferroptosis inducers in combination with conventional cancer therapies in various models [12–15].

Mitochondria are vital for adenosine triphosphate (ATP) production and utilize the electron transport chain (ETC), which is composed of four main complexes: complex I (NADH dehydrogenase), complex II (succinate dehydrogenase), complex III (cytochrome bc1 complex), and complex IV (cytochrome c oxidase). However, ATP production also produces ROS such as superoxide radicals and hydrogen peroxide as byproducts [7, 16]. Therefore, mitochondria, which play an essential role in maintaining the redox balance, could act as significant contributors to ROS production in the ferroptosis regulation.

Voltage-dependent anion channel 1 (VDAC1) is an abundant protein in the OMM that plays a gatekeeping role in the mitochondria-cytosol transport of metabolites [17]. VDAC1 facilitates the passive transport of ATP/ADP, calcium ions, fatty acids, and respiratory substrates across the OMM [18]. Downregulation of the VDAC1 channel diminishes metabolite exchange between the mitochondria and cytosol, resulting in the inhibition of cell growth [16, 19]. This indicates that VDAC1 plays a critical role in sustaining physiological and cellular functions. VDAC1 inhibition alleviates ferroptosis by protecting mitochondria in acetaminophen-induced acute liver injury and Aβ1-42-induced Alzheimer’s disease in mice [20]. However, the role of VDAC1 oligomerization in cysteine deprivation-induced ferroptosis remains unclear.

In the present study, we demonstrated that mitochondrial ROS production via the complex III electron transport chain and VDAC1 oligomerization play pivotal roles in cysteine deprivation-induced ferroptosis in various cancer cells. Through a comprehensive exploration of the role of each ETC complex in ROS production and the mechanisms underlying ferroptosis-induced mitochondrial perturbations, we aimed to understand the ferroptotic cell death pathways. Ultimately, our findings unveil novel therapeutic strategies for targeting dysregulated cell death pathways with potential implications for the treatment of diverse pathological conditions.

## Materials and methods

### Cell culture and cysteine deprivation

Human NSCLC cell line H1299, human breast cancer cell line MDA-MB-231, and human ovarian cancer cell line HEYA8 were obtained from the American Type Culture Collection (ATCC; Manassas, VA, USA). H1299 and HEYA8 cells were cultured in Roswell Park Memorial Institute (RPMI) 1640 (#LM011-01, Welgene, Gyeongsangbuk-do, Republic of Korea) and MDA-MB-231 cells were cultured in Dulbecco’s modified Eagle’s medium (DMEM; #LM001-05, Welgene) at 37 °C in 5% CO_2_. The medium was supplemented with 10% fetal bovine serum (FBS; #16000-044, Gibco; Thermo Fisher Scientific, Waltham, MA, USA).

Cysteine-free RPMI 1640 (#LM011-136) and DMEM media (#LM001-206) were custom-made by Welgene. During the experiments conducted under cysteine deprivation conditions, both the cysteine-free media and their corresponding control media were supplemented with 10% dialyzed fetal bovine serum (#26400-044, Gibco; Thermo Fisher Scientific), which lacked small molecules.

### Reagents

Ferrostatin-1 (#S7243), Liproxstatin-1 (#S7699), Deferoxamine mesylate (#S5742), and MitoQ mesylate (#S8978) were purchased from Selleck Chemicals (Houston, TX, USA). *N*-acetyl-l-cysteine (NAC; #A7250), MitoTEMPO (MitoT; #SML0737), Rotenone (#R8875), Diethyl butylmalonate (DBM; #112038), Antimycin A (#A8674), and Sodium azide (NaN_3_; #S2002) were obtained from Sigma-Aldrich (Merck KGaA, Darmstadt, Germany). CCCP (#M34152) was purchased from Thermo Fisher Scientific. DIDS sodium salt (#HY-D0086) and NSC15364 (#HY-108937) were obtained from MedChemExpress (Monmouth Junction, NJ, USA). BAPTA-AM (#B1205) was purchased from Invitrogen (Thermo Fisher Scientific).

### Cell viability analysis

Cell viability was determined by measuring the mitochondrial conversion of thiazolyl blue tetrazolium bromide (MTT; #M2128, Sigma-Aldrich; Merck KGaA). The amount of MTT formazan converted was calculated by measuring the absorbance at 570 nm. All experiments were performed in triplicate, and cell viability (%) was expressed as a percentage relative to the control cells.

### Transient transfection

The human VDAC1 gene was cloned into eukaryotic expression plasmids pEGFP-C1 and pCMV Tag 2B, which encode GFP and Flag, respectively. VDAC1 (#sc-42355) and control (CTL; #sc-37007) small interfering RNAs (siRNAs) were purchased from Santa Cruz Biotechnology (Dallas, TX, USA). Transfection with plasmids and siRNAs was performed using the Lipofectamine 3000 transfection reagent (#L3000015, Invitrogen; Thermo Fisher Scientific) and Lipofectamine RNAiMAX (#13778, Invitrogen; Thermo Fisher Scientific), respectively, according to the manufacturer’s instructions.

### Western blot analysis

Western blotting was performed as previously described [21]. Briefly, protein samples were separated using 10–12% sodium dodecyl sulfate-polyacrylamide gels and transferred to nitrocellulose membranes, followed by immunoblotting with specific primary and horseradish peroxidase-conjugated secondary antibodies. The following antibodies were used: anti-GFP (#sc-9996) obtained from Santa Cruz Biotechnology and anti-β-actin (#A5441) obtained from Sigma-Aldrich (Merck KGaA).

### Quantitative real-time PCR

RNA extraction and cDNA synthesis were performed as previously described [22]. Quantitative real-time PCR was conducted using FastStart Essential DNA Green Master (#06924204001, Roche, Basel, Switzerland) on a Lightcycle96 real-time PCR system (Roche). The following primers were used: VDAC1 (5’- CGGGCAGTCTGGAAACCAAG-3’ and 5’-CGAAGGTCAGCTTCAGTCC-3’, 135 bp product) and β-actin (5’- CACCATTGGCAATGAGCGGTTC-3’ and 5’-AGGTCTTTGCGGATGTCCACGT-3’, 135 bp product) [23]. Relative gene expression level was determined using the 2^–ΔΔ*CT*^ method.

### Flow cytometry analysis

Cells were cultured overnight in a 6-well plate to reach approximately 40–50% confluence. After treatment, the cells were incubated for 30 min at 37 °C with the following fluorescence sensors: 2 μM C11-BODIPY (#D3861, Invitrogen; Thermo Fisher Scientific), as a lipid peroxidation sensor; 1 μM FerroOrange (#F374, Dojindo, Kumamoto, Japan), as an intracellular Fe^2+^ sensor; 100 nM MitoPeDPP (#M466, Dojindo), as a mitochondrial lipid peroxide detector; 2.5 μM MitoSOX (#M36008, Invitrogen; Thermo Fisher Scientific), as a mitochondrial superoxide indicator; and 100 nM Tetramethylrhodamine ethyl ester perchlorate (TMRE, #HY-D0985A, MedChemExpress), as a mitochondrial membrane potential sensor. The cell pellets were collected in Hank’s balanced salt solution. A total of 5000 cells were analyzed using a CytoFLEX flow cytometer (#A00-1-1102, Beckman Coulter, Brea, CA, USA). Data from dead cells and cell debris signals were excluded from the analysis.

### Immunofluorescence imaging

Cells were seeded at a density of 1.0 × 10^6^ cells/well onto coverslips in a 6-well plate and grown overnight until approximately 50% cell confluence. Following treatment, the cells were stained with the mitochondrial ROS sensor MitoSOX (#M36008, Invitrogen; Thermo Fisher Scientific) and mitochondrial dye MitoBright LT Green (#MT10, Dojindo) for 30 min at 37 °C, and subsequently fixed with 4% paraformaldehyde for 15 min at room temperature. The cells were visualized using a Zeiss Axio Imager-M2 fluorescent microscope (Carl Zeiss, Jena, Germany) equipped with an Axiocam 506 camera.

### Oxygen consumption measurement

Oxygen consumption in cells was measured using the extracellular oxygen consumption assay kit (#ab197243; Abcam, Cambridge, UK) according to the manufacturer’s instructions. Briefly, 1.0 × 10^4^ cells were seeded in 96-well clear-bottom black plates and incubated overnight. Following treatment, the reconstituted extracellular O_2_ consumption reagent was added to each sample well, which was promptly sealed with mineral oil. Fluorescence intensities were measured using a SpectraMax i3x microplate reader (Molecular Devices, Sunnyvale, CA, USA) at Ex/Em = 380/650 nm, with measurements taken at 1.5 min intervals over a total duration of 180 min, and fluorescence intensity was normalized to the intensity of the blank, using fresh culture medium as the blank control.

### Statistical analysis

The data are expressed as the mean ± standard deviation (SD) of three independent experiments. Differences were analyzed by one-way ANOVA followed by Tukey’s test using GraphPad Prism software (version 8.0, San Diego, CA, USA). Differences were considered statistically significant at *p* < 0.05.

## Results

### Cysteine deprivation induces ferroptosis

**Fig. 1:**
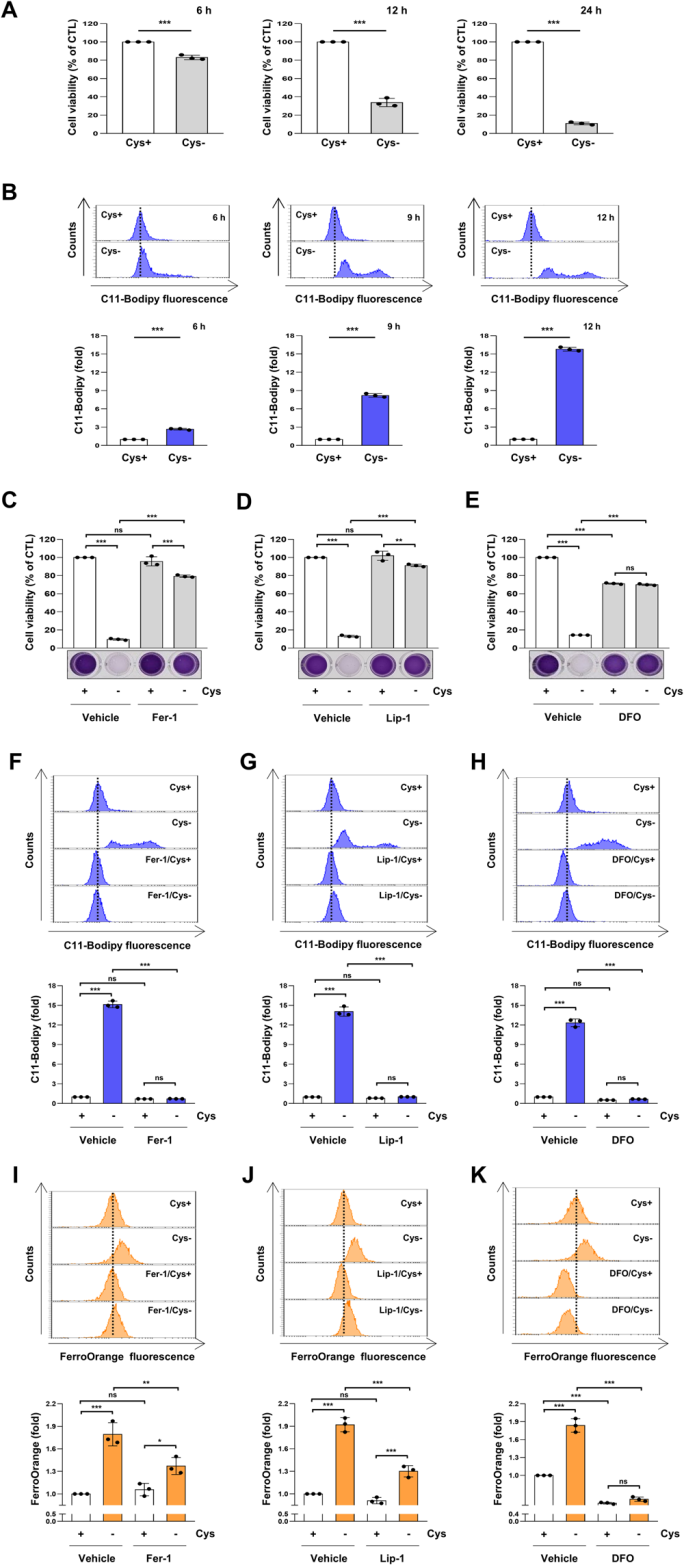
Cysteine deprivation-induced ferroptosis. **A**, **B** H1299 cells were incubated in a cysteine-deprived medium for the indicated time. **C**–**E** H1299 cells were incubated in a control or cysteine-deprived medium with or without 2.5 μM Fer-1, 200 nM Lip-1, or 50 μM DFO for 24 h. **A**, **C**–**E** Cell viability was measured by MTT assay, (*n* = 3; **p < 0.01, ***p < 0.001, ns not significantly different). **F**–**K** H1299 cells were incubated in a control or cysteine-deprived medium with or without 2.5 μM Fer-1, 200 nM Lip-1, or 50 μM DFO for 12 h. **B**, **F**–**H** A representative flow cytometry histogram of C11-BODIPY staining (upper panels) and the average MFI ± SD of C11-BODIPY staining (low panels) (*n* = 3; ***p < 0.001, ns not significantly different). **I**–**K** A representative flow cytometry histogram of FerroOrange staining (upper panels) and the average MFI ± SD of FerroOrange staining (low panels) (*n* = 3; *p < 0.05, **p < 0.01, ***p < 0.001, ns not significantly different). Cys cysteine, DFO deferoxamine mesylate, Fer-1 ferrostatin-1, Lip-1 liproxstatin-1, MFI mean fluorescence intensity, SD standard deviation.

To investigate whether cysteine deprivation induces ferroptosis, we depleted cysteine in H1299 cells at various time points and detected ferroptosis by measuring lipid peroxidation levels using the C11-BODIPY probe. As shown in Fig. 1A, B, cysteine deprivation resulted in a time-dependent decrease in cell viability and a corresponding increase in lipid peroxidation levels. Subsequently, we examined whether the ferroptosis inhibitors, Ferrostatin-1 (Fer-1) or Liproxstatin-1 (Lip-1), or the iron chelator Deferoxamine (DFO), attenuated ferroptosis induction resulting from cysteine deprivation. Treatment with Fer-1, Lip-1, or DFO significantly restored reduced cell viability and alleviated increased lipid peroxidation induced by cysteine deprivation (Fig. 1C–H). Additionally, when observing labile iron levels using the FerroOrange probe, they also inhibited labile iron accumulation induced by cysteine deprivation (Fig. 1I–K). These findings suggest that cysteine deprivation induces ferroptosis.

### Cysteine deprivation induces ferroptosis through mitochondrial ROS production

**Fig. 2:**
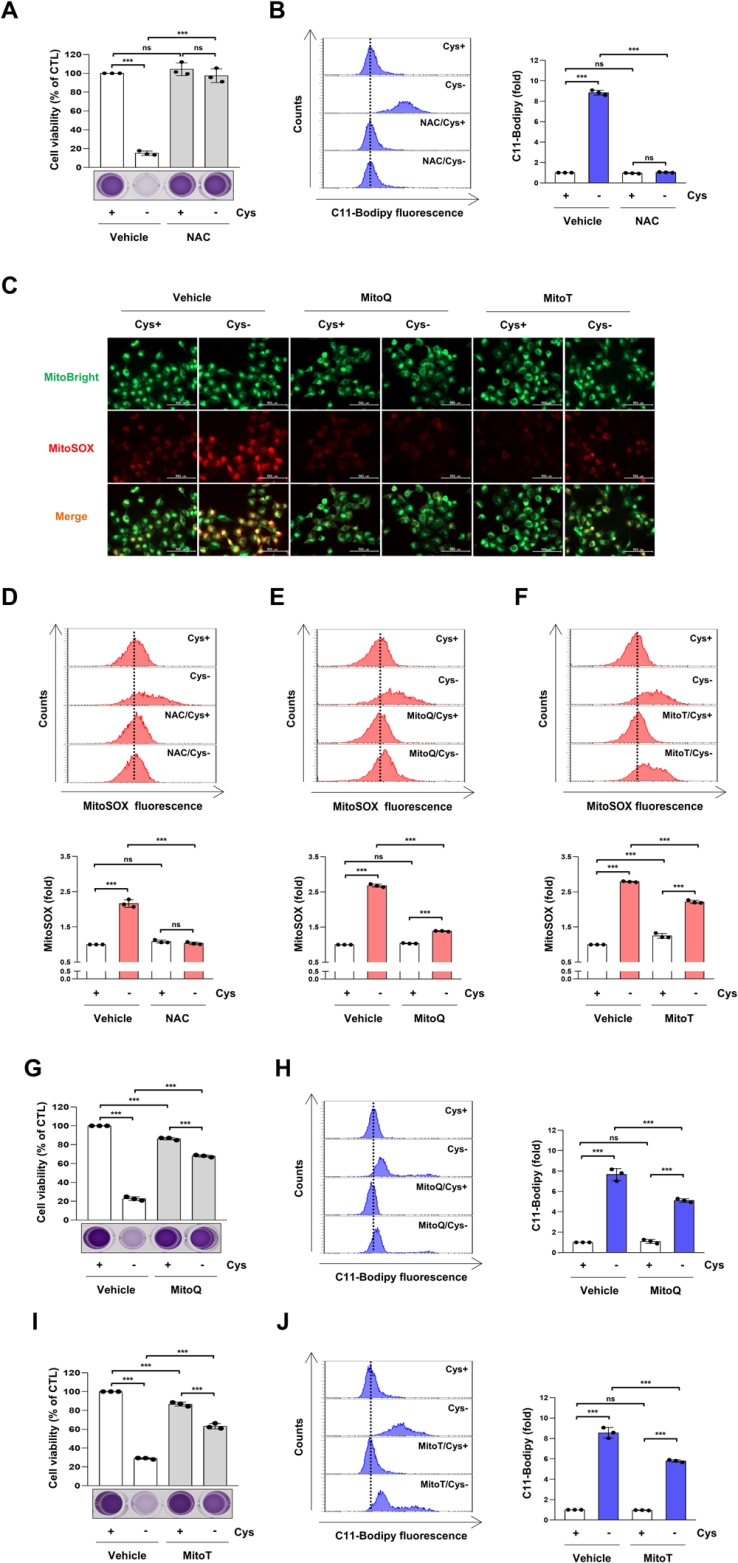
Mitochondrial ROS scavengers block ferroptosis. **A** H1299 cells were incubated in a control or cysteine-deprived medium with or without 5 mM NAC for 24 h. **B** H1299 cells were incubated in a control or cysteine-deprived medium with or without 5 mM NAC for 12 h. **C** H1299 cells were incubated in a control or cysteine-deprived medium with or without 5 mM NAC, 1 μM MitoQ (ubiquinone derivative), or 200 μM MitoT (superoxide dismutase mimetic) for 12 h. A representative fluorescence microscopy image of MitoSOX red staining. The green fluorescence of MitoBright LT green shows the location of mitochondria (Scale bar = 100 μm). **D**–**F** H1299 cells were incubated in a control or cysteine-deprived medium with or without 5 mM NAC, 1 μM MitoQ, or 200 μM MitoT for 12 h. A representative flow cytometry histogram of MitoSOX staining (upper panels) and the average MFI ± SD of MitoSOX staining (low panels) (*n* = 3; ***p < 0.001, ns not significantly different). **G**, **I** H1299 cells were incubated in a control or cysteine-deprived medium with or without 1 μM MitoQ or 200 μM MitoT for 24 h. **A**, **G**, and **I** Cell viability was measured by MTT assay (*n* = 3; ***p < 0.001, ns not significantly different). **H**, **J** H1299 cells were incubated in a control or cysteine-deprived medium with or without 1 μM MitoQ or 200 μM MitoT for 12 h. **B**, **H**, and **J** A representative flow cytometry histogram of C11-BODIPY staining (left panels) and the average MFI ± SD of C11-BODIPY staining (right panels) (*n* = 3; ***p < 0.001, ns not significantly different). Cys cysteine, MFI mean fluorescence intensity, MitoQ MitoQuinone, MitoT MitoTEMPO, NAC *N*-acetyl cysteine, SD standard deviation.

The accumulation of reactive oxygen species (ROS) within mitochondria has been reported to induce ferroptosis [24]. Thus, we subsequently investigated the involvement of ROS in ferroptosis induction by cysteine deprivation. Treatment with *N*-acetylcysteine (NAC), a ROS scavenger, improved cell viability, and suppressed lipid peroxidation in cysteine-deprived H1299 cells (Fig. 2A, B). As shown in Fig. 2C, D, cysteine deprivation increased MitoSOX fluorescence, indicating ROS accumulation within the mitochondria. NAC treatment effectively suppressed mitochondrial ROS accumulation induced by cysteine deprivation (Fig. 2D). To further investigate the role of mitochondrial ROS in cysteine deprivation-induced ferroptosis, cysteine-deprived H1299 cells were treated with mitochondrial ROS scavengers MitoQuinone (MitoQ) and MitoTEMPO (MitoT). MitoQ is a ubiquinone derivative, while MitoT is a superoxide dismutase mimetic. Both compounds are conjugated to the lipophilic triphenylphosphonium cation, enabling them to accumulate in the inner mitochondrial membrane, where they scavenge superoxide [25, 26]. The accumulation of mitochondrial ROS induced by cysteine deprivation was mitigated by MitoQ or MitoT (Fig. 2C, E, and F). Furthermore, treatment with MitoQ and MitoT ameliorated cell viability and attenuated lipid peroxidation in cysteine-deprived H1299 cells (Fig. 2G–J). Collectively, these findings suggest that the elevation of mitochondrial ROS levels due to cysteine deprivation induces ferroptosis.

### Cysteine deprivation induces ferroptosis via modulating mitochondria function

**Fig. 3:**
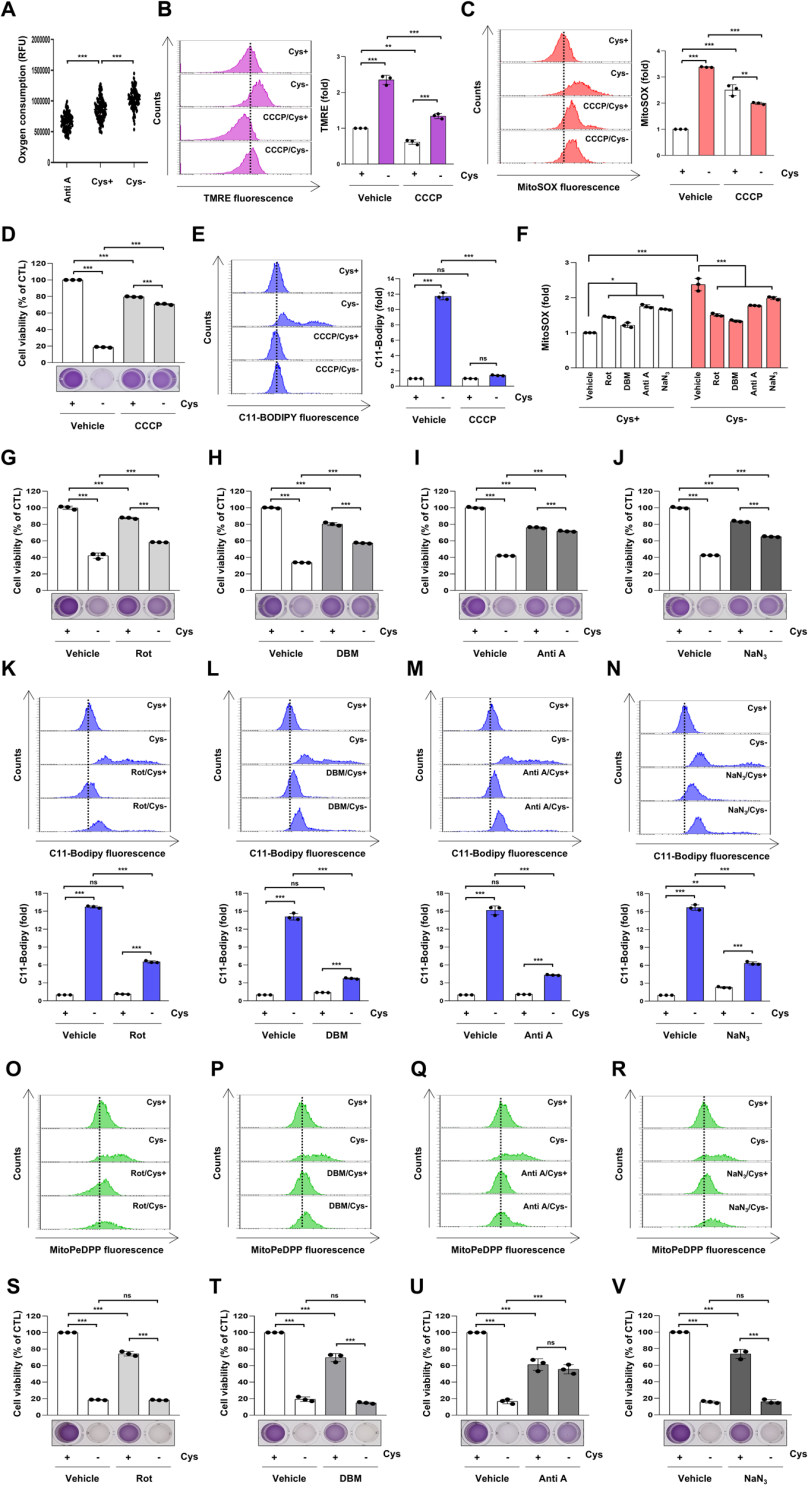
Inhibition of electron transport chain (ETC) activity blocks ferroptosis induced by cysteine deprivation. **A** H1299 cells were incubated in a control or cysteine-deprived medium for 2 h. The oxygen consumption was measured using an extracellular oxygen consumption assay. The dots represent the values of oxygen consumption measured at 1.5 min intervals over a period of 180 min (*n* = 3; ***p < 0.001). H1299 cells treated with 75 μM Antimycin A for 2 h were used as a positive control for oxygen consumption rate inhibition. **B**, **C**, and **E** H1299 cells were incubated in a control or cysteine-deprived medium with or without 5 μM CCCP for 12 h. **B** A representative flow cytometry histogram of TMRE staining (left panel) and the average MFI ± SD of TMRE staining (right panel) (*n* = 3; **p < 0.01, ***p < 0.001). **C** A representative flow cytometry histogram of MitoSOX staining (left panel) and the average MFI ± SD of MitoSOX staining (right panel) (*n* = 3; **p < 0.01, ***p < 0.001). **D** H1299 cells were incubated in a control or cysteine-deprived medium with or without 5 μM CCCP for 24 h. **F–R** H1299 cells were incubated in control or cysteine-deprived medium with or without 200 nM Rotenone (complex I inhibitor), 1.5 mM DBM (complex II inhibitor), 75 μM Antimycin A (complex III inhibitor), or 1.5 mM NaN_3_ (complex IV inhibitor) for 12 h. **F** A representative flow cytometry of the average MFI ± SD of C11-BODIPY staining (*n* = 3; *p < 0.05, ***p < 0.001). **E**, **K**–**N** A representative flow cytometry histogram of C11-BODIPY staining (left or upper panels) and the average MFI ± SD of C11-BODIPY staining (right or low panels) (*n* = 3; **p < 0.01, ***p < 0.001, ns not significantly different). **O**–**R** A representative flow cytometry histogram of MitoPeDPP staining. **S**–**V** H1299 cells were incubated in a control or cysteine-deprived medium with or without 200 nM Rotenone, 1.5 mM DBM, 75 μM Antimycin A, or 1.5 mM NaN_3_ for 24 h. **D**, **G**–**J**, and **S**–**V** Cell viability was measured by MTT assay (*n* = 3; ***p < 0.001, ns not significantly different). Anti A antimycin A, Cys cysteine, DBM diethyl butylmalonate, NaN_3_ sodium azide, MFI mean fluorescence intensity, Rot rotenone, SD standard deviation, TMRE tetramethylrhodamine ethyl ester perchlorate.

Given the importance of mitochondria in ROS production and ferroptosis induction, we investigated the effect of cysteine deprivation on mitochondrial functions, including mitochondrial respiration and mitochondrial membrane potential (MMP). As shown in Fig. 3A, mitochondrial respiration, as measured by oxygen consumption, increased in response to cysteine deprivation. Cysteine-deprived cells showed a dramatic increase in TMRE fluorescence, indicating MMP hyperpolarization (Fig. 3B). Treatment with the CCCP, mitochondrial oxidative phosphorylation uncoupler, attenuates the increase in MMP and ROS levels induced by cysteine deprivation (Fig. 3B, C). CCCP restored reduced cell viability and decreased elevated lipid peroxidation under cysteine deprivation (Fig. 3D, E), suggesting that MMP hyperpolarization is associated with cysteine deprivation-induced ferroptosis. To further investigate the role of electron transport chain (ETC) activity in ferroptosis induced by cysteine deprivation, H1299 cells deprived of cysteine were treated for 12 h with inhibitors of mitochondrial complex I (Rotenone), complex II (DBM), complex III (Antimycin A), or complex IV (NaN_3_). As shown in Fig. 3F–N, these inhibitors increased mitochondrial ROS and decreased cell viability by 20–30%, but did not induce lipid peroxidation, thus not triggering ferroptosis. However, under cysteine deprivation, the inhibitors significantly reduced elevated mitochondrial ROS, restored reduced cell viability, and alleviated increased lipid peroxidation. Furthermore, the inhibitors suppressed mitochondrial lipid peroxidation in cysteine-deprived cells, as detected by MitoPeDPP (Fig. 3O–R). These findings suggest that inhibiting ETC activity under cysteine deprivation reduces ROS and mitigates ferroptosis. After 24 h of treatment, only Antimycin A significantly alleviated the cysteine deprivation-induced reduction in cell viability (Fig. 3S–V). These results indicate that while all four ETC complexes contribute to ferroptosis induced by cysteine deprivation, complex III plays a more critical role.

### Inhibition of VDAC1 oligomerization suppresses cysteine deprivation-induced ferroptosis

**Fig. 4:**
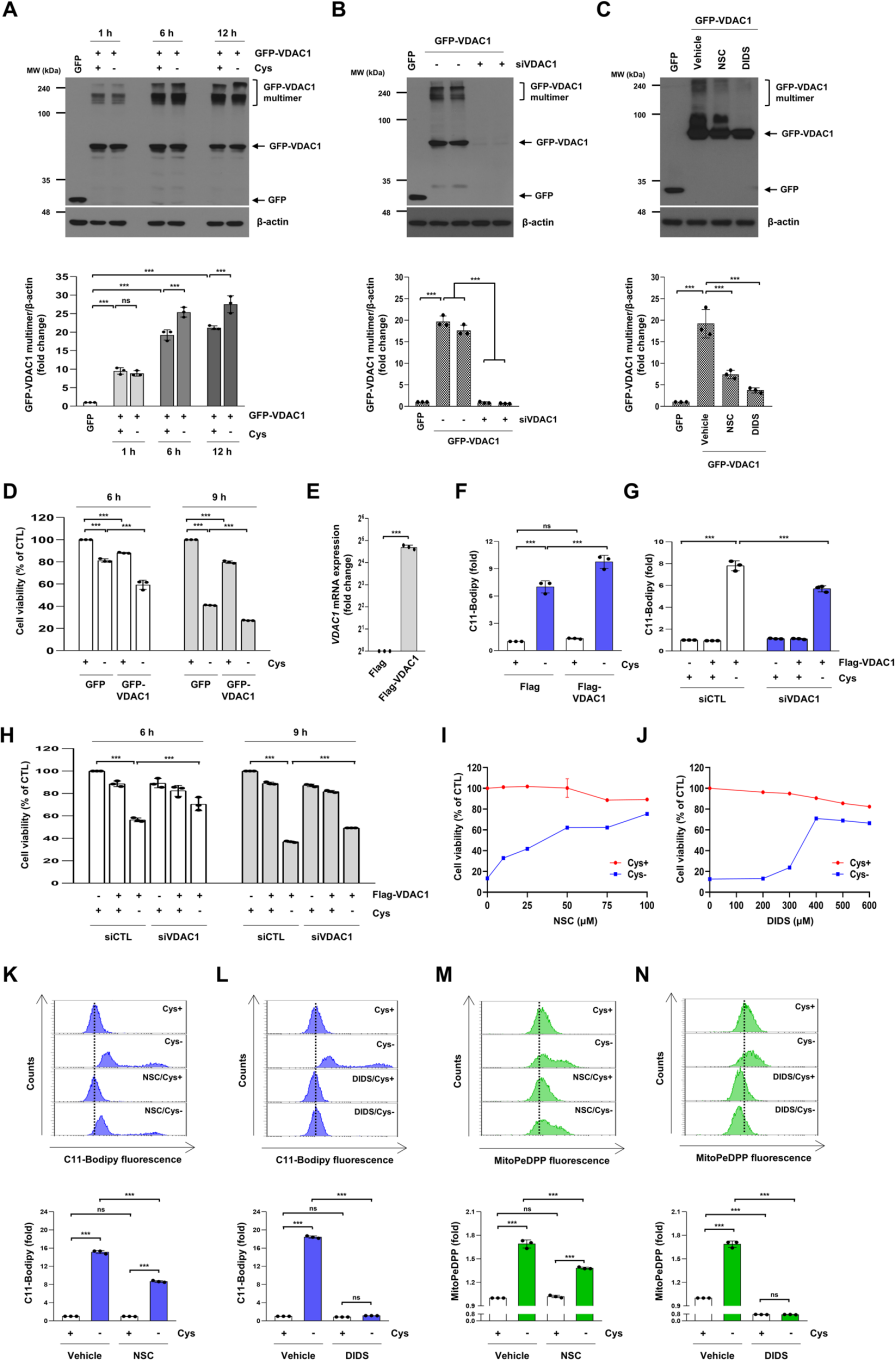
Inhibition of VDAC1 oligomerization suppresses ferroptosis. **A** H1299 cells were transfected with GFP or GFP-VDAC1 for 18 h and subsequently treated with a control or cysteine-deprived medium for the indicated time. **B** H1299 cells were co-transfected with CTL or VDAC1 siRNAs along with GFP or GFP-VDAC1 for 24 h. **C** H1299 cells were transfected with GFP or GFP-VDAC1 for 18 h and subsequently treated with 100 μM NSC15364 or 400 μM DIDS for 12 h. **A**–**C** The indicated protein levels were estimated by western blot analysis. Band intensities of GFP-VDAC1 were quantified using Image (**J**), normalized to β-actin intensity, and plotted as a fold change relative to the control. **D** H1299 cells were transfected with GFP or GFP-VDAC1 for 18 h and subsequently treated with a control or cysteine-deprived medium for the indicated time. **E** H1299 cells were transfected with Flag or Flag-VDAC1 for 24 h. The indicated mRNA levels were estimated by quantitative real-time PCR analysis (*n* = 3; ***p < 0.001). **F** H1299 cells were transfected with Flag or Flag-VDAC1 for 18 h and subsequently treated with control or cysteine-deprived medium for 9 h. **G**, **H** H1299 cells were co-transfected with CTL or VDAC1 siRNAs along with Flag or Flag-VDAC1 for 18 h and subsequently treated with control or cysteine-deprived medium for the indicated time (**H**) or 9 h (**G**). **I**, **J** H1299 cells were incubated in a control or cysteine-deprived medium with or without NSC15364 or DIDS for the indicated concentration for 24 h. **D**, **H**, **I**, and **J** Cell viability was measured by MTT assay (*n* = 3; ***p < 0.001). **K**–**N** H1299 cells were incubated in a control or cysteine-deprived medium with or without 100 μM NSC15364 or 400 μM DIDS for 12 h. **F**, **G** A representative flow cytometry of the average MFI ± SD of C11-BODIPY staining (*n* = 3; ***p < 0.001, ns not significantly different). **K**, **L** A representative flow cytometry histogram of C11-BODIPY staining (upper panels) and the average MFI ± SD of C11-BODIPY staining (low panels) (*n* = 3; ***p < 0.001, ns not significantly different). **M**, **N** A representative flow cytometry histogram of MitoPeDPP staining (upper panels) and the average MFI ± SD of MitoPeDPP staining (low panels) (*n* = 3; ***p < 0.001, ns not significantly different). CTL control, Cys cysteine, MFI mean fluorescence intensity, NSC NSC15364, SD standard deviation.

Voltage-dependent anion channel 1 (VDAC1), ubiquitously present in the outer mitochondrial membrane (OMM), is a key regulator of mitochondrial function [27]. Overexpression of VDAC1 promotes its oligomerization, resulting in the formation of large pores in the OMM and ultimately leading to cell death [28]. Ectopic expression of GFP-VDAC1 in H1299 cells led to a time-dependent increase in GFP-VDAC1 oligomer formation, as evidenced by the elevated molecular weight bands corresponding to its multimeric state and reduced cell viability by 15–20% (Fig. 4A, D). This oligomerization was reduced by siRNA specifically targeting VDAC1 (Fig. 4B). NSC15364 and DIDS are known to directly interact with VDAC1 to prevent its oligomerization [29, 30]. As shown in Fig. 4C, these two inhibitors also significantly decreased VDAC1 oligomerization by over 60%. A recent study demonstrated that VDAC1 oligomerization is associated with abnormal iron metabolism, mitochondrial damage, and ferroptosis in hepatocytes [31]. We further investigated whether cysteine deprivation increases VDAC1 oligomerization. GFP-VDAC1 was overexpressed in H1299 cells, followed by cysteine deprivation for 1 h, 6 h, or 12 h. While 1 h of cysteine deprivation did not affect VDAC1 oligomerization, a significant increase in VDAC1 oligomerization was observed after 6 h and 12 h of cysteine deprivation (Fig. 4A). Moreover, cysteine deprivation further reduced cell viability in cells overexpressing VDAC1 (Fig. 4D). In cells overexpressing Flag-VDAC1, an increase in VDAC1 expression at the mRNA level was observed (Fig. 4E), and cysteine deprivation was found to further enhance lipid peroxidation in these cells (Fig. 4F). Treatment with VDAC1 siRNA in VDAC1 overexpressing cells alleviated the decrease in cell viability and the increase in lipid peroxidation caused by cysteine deprivation (Fig. 4G, H). The VDAC1 oligomerization inhibitors NSC15364 and DIDS prevented the decrease in cell viability caused by cysteine deprivation in a dose-dependent manner (Fig. 4I, J). Also, NSC15364 and DIDS suppressed the increase in lipid peroxidation and mitochondrial lipid peroxidation in cysteine-deprived cells (Fig. 4K–N). These results suggest that VDAC1 oligomerization may contribute to the mediation of ferroptosis induced by cysteine deprivation.

**Fig. 5:**
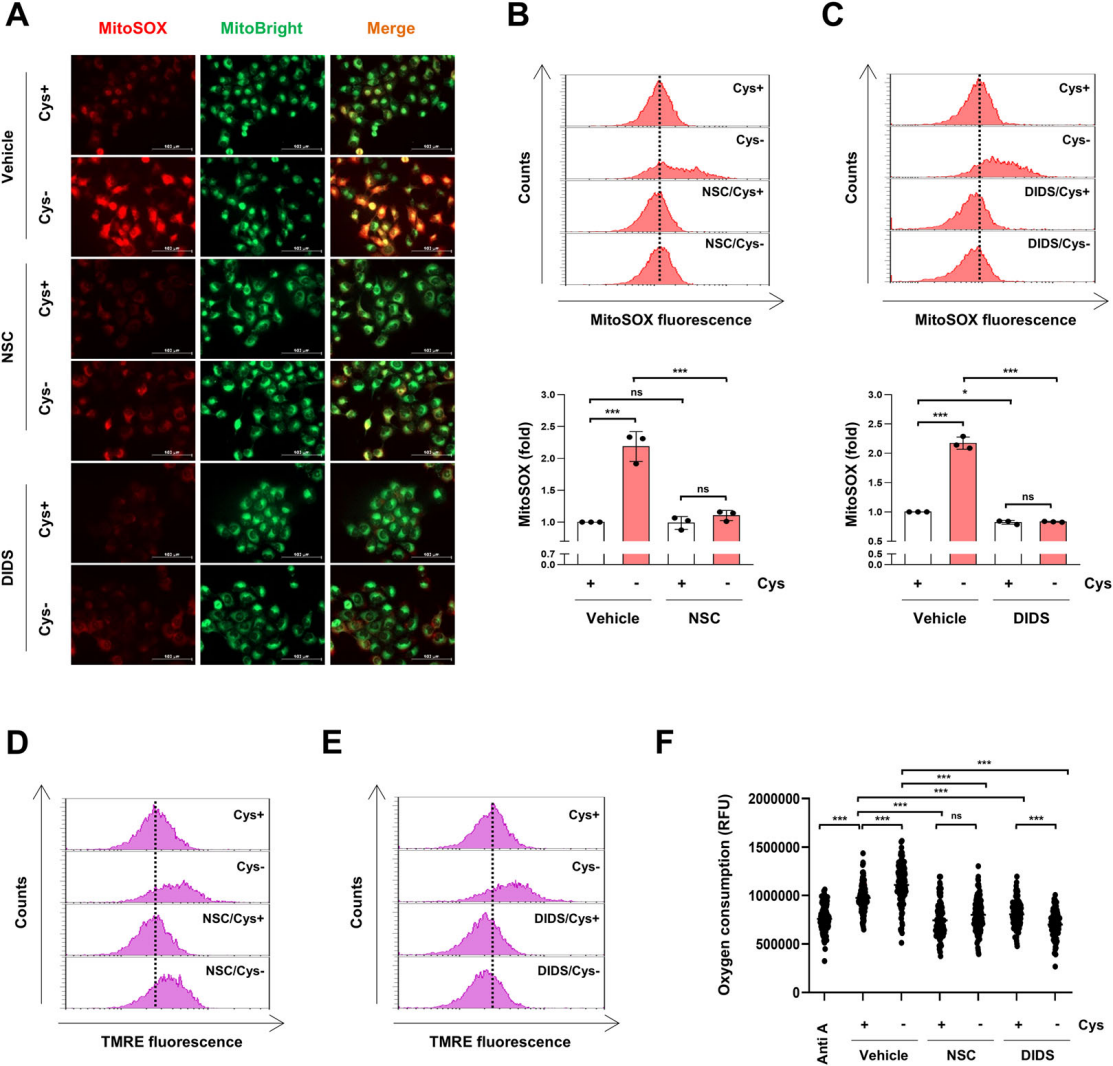
Inhibition of VDAC1 oligomerization blocks cysteine deprivation-induced ferroptosis via mitochondrial ROS suppression. **A**–**E** H1299 cells were incubated in a control or cysteine-deprived medium with or without 100 μM NSC15364 or 400 μM DIDS for 12 h. **A** A representative fluorescence microscopy image of MitoSOX red staining. The green fluorescence of MitoBright LT green shows the location of mitochondria (scale bar = 100 μm). **B**, **C** A representative flow cytometry histogram of MitoSOX staining (upper panels) and the average MFI ± SD of MitoSOX staining (low panels) (*n* = 3; *p < 0.05, ***p < 0.001, ns not significantly different). **D**, **E** A representative flow cytometry histogram of TMRE staining. **F** H1299 cells were incubated in a control or cysteine-deprived medium with or without 100 μM NSC15364 or 400 μM DIDS for 2 h. The oxygen consumption was measured using an extracellular oxygen consumption assay. The dots represent the values of oxygen consumption measured at 1.5-min intervals over a period of 180 min. H1299 cells treated with 75 μM Antimycin A for 2 h were used as a positive control for oxygen consumption rate inhibition, (*n* = 3; ***p < 0.001, ns not significantly different). Anti A antimycin A, Cys cysteine, MFI mean fluorescence intensity, NSC NSC15364, SD standard deviation, TMRE tetramethylrhodamine ethyl ester perchlorate.

Interestingly, NSC15364 and DIDS blocked mitochondrial ROS accumulation under cysteine deprivation (Fig. 5A–C). Furthermore, NSC15364 and DIDS inhibited cysteine deprivation-induced TMRE fluorescence intensity, suggesting that these inhibitors suppress MMP hyperpolarization caused by cysteine deprivation (Fig. 5D, E). These inhibitors suppressed oxygen consumption to a similar extent as the complex III inhibitor Antimycin A and inhibited the oxygen consumption induced by cysteine deprivation (Fig. 5F). These data suggest that VDAC1 inhibition reverses the mitochondrial dysfunction induced by cysteine deprivation, thereby alleviating ferroptosis.

### Cysteine deprivation-induced ferroptosis is not dependent on calcium

**Fig. 6:**
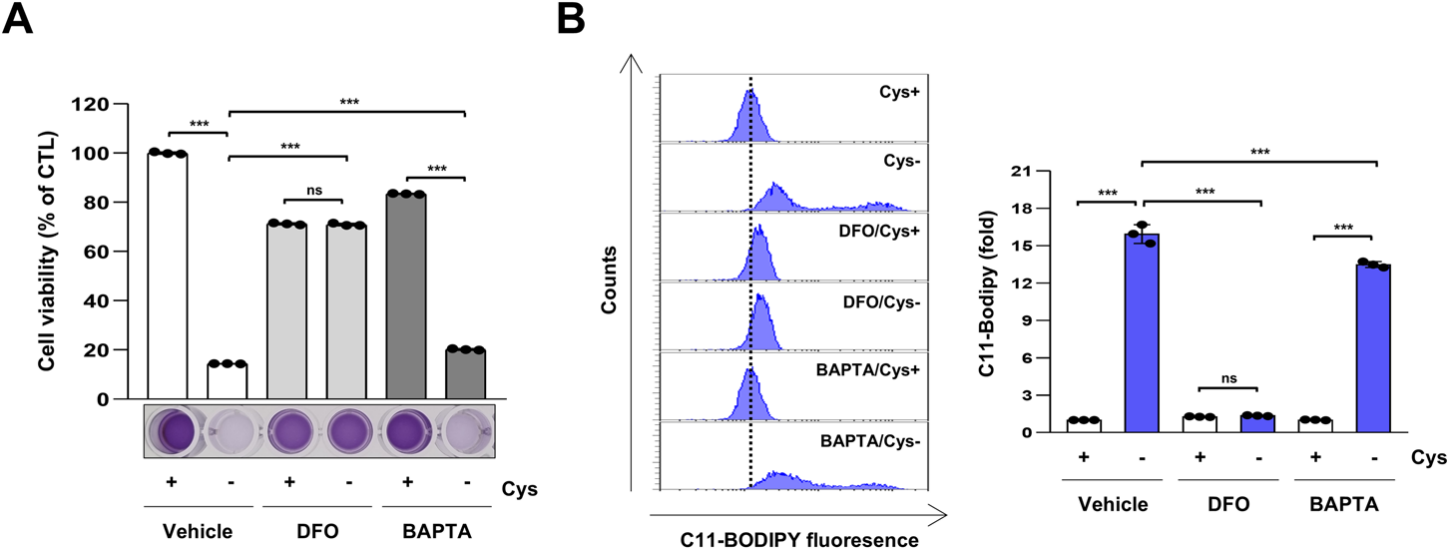
Cysteine deprivation-induced ferroptosis is calcium-independent. **A** H1299 cells were incubated in a control or cysteine-deprived medium with or without 50 μM DFO or 5 μM BAPTA for 24 h. Cell viability was measured by MTT assay, (*n* = 3; ***p < 0.001, ns not significantly different). **B** H1299 cells were incubated in a control or cysteine-deprived medium with or without 50 μM DFO or 5 μM BAPTA for 12 h. A representative flow cytometry histogram of C11-BODIPY staining (left panel) and the average MFI ± SD of C11-BODIPY staining (right panel) (*n* = 3; ***p < 0.001). Cys cysteine, DFO deferoxamine, MFI mean fluorescence intensity, SD standard deviation.

VDAC1 possesses Ca^2+^ binding sites and transports Ca^2+^ into the mitochondria [32, 33]. Mitochondrial Ca^2+^ boosts mitochondrial respiration and induces apoptosis [34]. To investigate the involvement of Ca^2+^ in cysteine deprivation-induced ferroptosis, we treated cysteine-deprived H1299 cells with the calcium chelator BAPTA. The iron chelator DFO prevented cysteine deprivation-induced ferroptosis, whereas the calcium chelator BAPTA did not restore decreased cell viability or increased lipid peroxidation induced by cysteine deprivation (Fig. 6A, B). These findings suggest that cysteine deprivation-induced ferroptosis is not calcium-dependent.

### Inhibition of VDAC1 oligomerization suppresses ferroptosis induced by RSL3

**Fig. 7:**
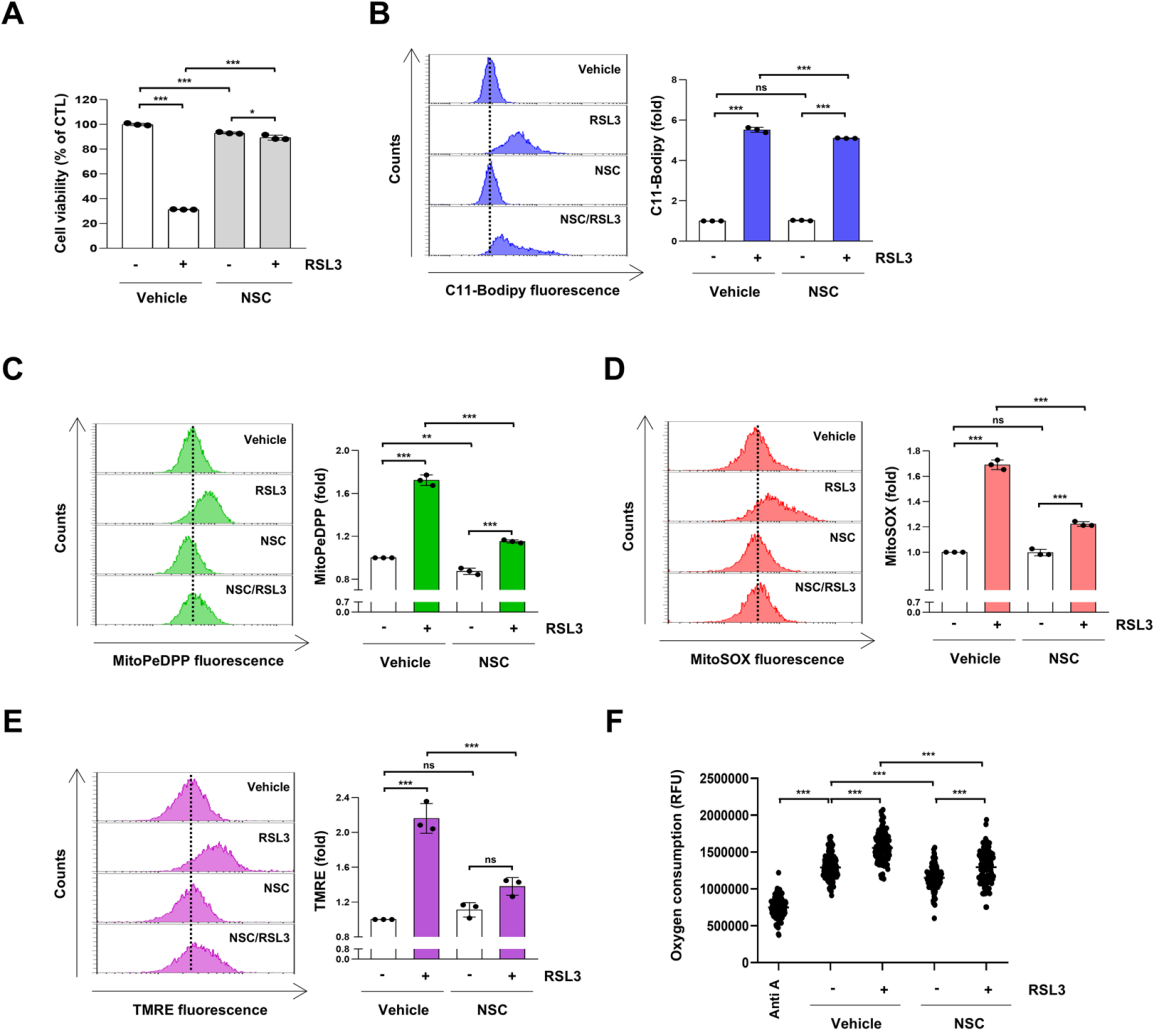
Inhibition of VDAC1 oligomerization blocks RSL3-mediated ferroptosis. **A** H1299 cells were treated with 200 nM RSL3 and 100 μM NSC15364 for 18 h. Cell viability was measured by MTT assay, (*n* = 3; *p < 0.05, ***p < 0.001). **B**–**E** H1299 cells were treated with 200 nM RSL3 and 100 μM NSC15364 for 9 h. **B** A representative flow cytometry histogram of C11-BODIPY staining (left panel) and the average MFI ± SD of C11-BODIPY staining (right panel) (*n* = 3; ***p < 0.001, ns not significantly different). **C** A representative flow cytometry histogram of MitoPeDPP staining (left panel) and the average MFI ± SD of MitoPeDPP staining (right panel) (*n* = 3; **p < 0.01, ***p < 0.001). **D** A representative flow cytometry histogram of MitoSOX staining (left panel) and the average MFI ± SD of MitoSOX staining (right panel) (*n* = 3; ***p < 0.001, ns not significantly different). **E** A representative flow cytometry histogram of TMRE staining (left panel) and the average MFI ± SD of TMRE staining (right panel) (*n* = 3; ***p < 0.001, ns not significantly different). **F** H1299 cells were treated with 200 nM RSL3 and 100 μM NSC15364 for 2 h. The oxygen consumption was measured using an extracellular oxygen consumption assay. The dots represent the values of oxygen consumption measured at 1.5-min intervals over a period of 180 min. H1299 cells treated with 75 μM Antimycin A for 2 h were used as a positive control for oxygen consumption rate inhibition, (*n* = 3; ***p < 0.001). Anti A antimycin A, Cys cysteine, MFI mean fluorescence intensity, NSC NSC15364, SD standard deviation, TMRE tetramethylrhodamine ethyl ester perchlorate.

We investigated whether other ferroptosis inducers such as RSL3 also modulate VDAC1 to induce ferroptosis. RSL3 is a small molecule compound widely used as a ferroptosis inducer that specifically inhibits glutathione peroxidase 4 [35]. The VDAC1 oligomerization inhibitor, NSC15364, mitigated the decrease in H1299 cell viability induced by RSL3 (Fig. 7A). Furthermore, NSC15364 suppressed the increase in lipid peroxidation in H1299 cells treated with RSL3 (Fig. 7B). Additionally, NSC15364 reduced the increase in mitochondrial lipid peroxidation and mitochondrial ROS in H1299 cells exposed to RSL3 (Fig. 7C, D). Similar to cysteine-deprived conditions, we observed an increase in MMP and oxygen consumption in H1299 cells treated with RSL3 (Fig. 7E, F). Treatment with NSC15364 alleviated MMP hyperpolarization and increased the oxygen consumption caused by RSL3 (Fig. 7E, F). These results suggest that inhibition of VDAC1 oligomerization reduced ferroptosis induced by RSL3.

### Inhibition of VDAC1 oligomerization suppresses cysteine deprivation-induced ferroptosis in breast and ovarian cancer cells

**Fig. 8:**
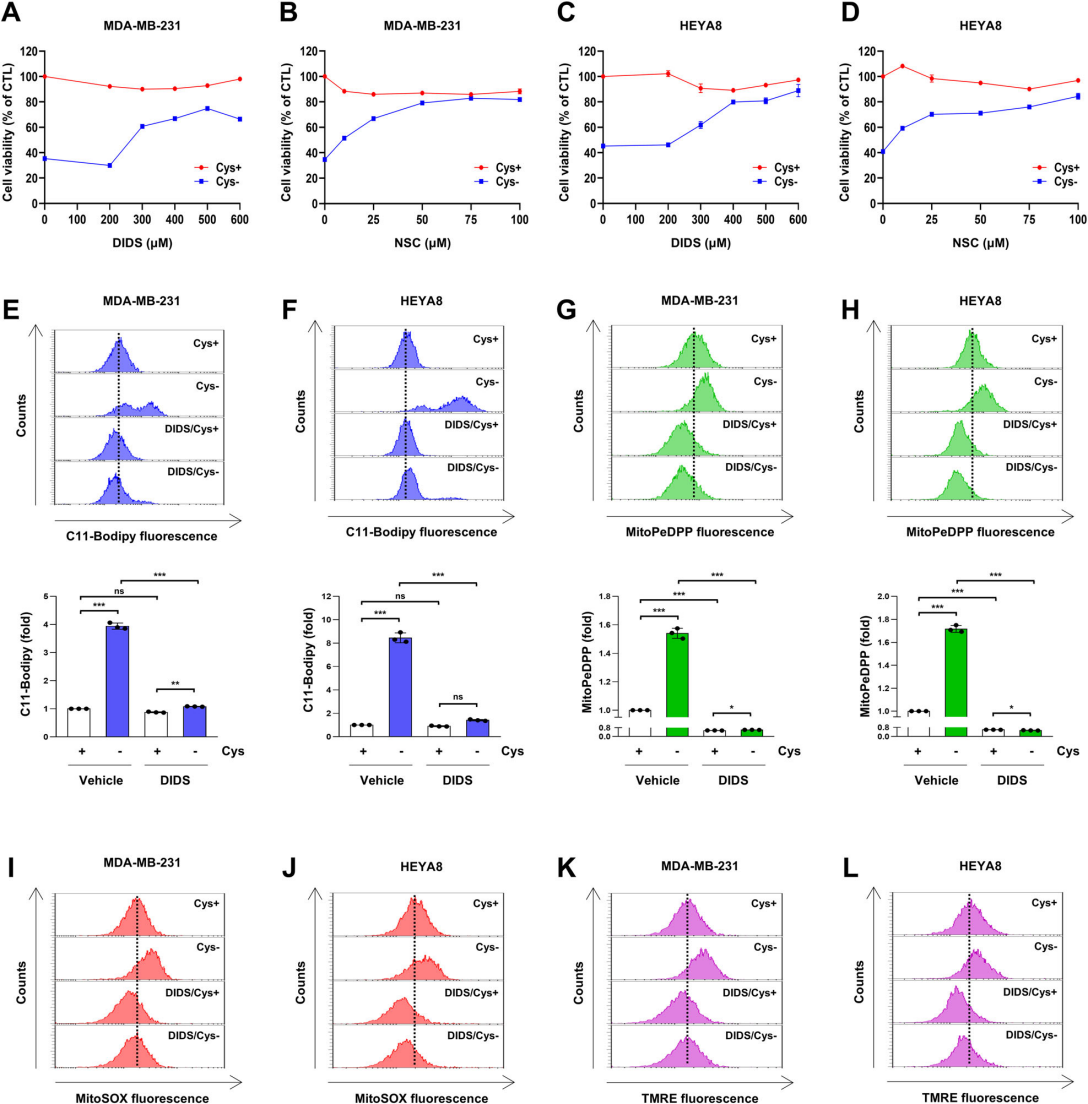
Inhibition of VDAC1 oligomerization suppresses ferroptosis in various cell lines. **A**, **B** MDA-MB-231 cells were incubated in a control or cysteine-deprived medium with or without NSC15364 or DIDS for the indicated concentration for 24 h. **C**, **D** HEYA8 cells were incubated in a control or cysteine-deprived medium with or without NSC15364 or DIDS for the indicated concentration for 24 h. **A**–**D** Cell viability was measured by MTT assay (*n* = 3). **E**, **G**, **I**, and **K** MDA-MB-231 cells were incubated in a control or cysteine-deprived medium with or without 100 μM NSC15364 or 400 μM DIDS for 12 h. **F**, **H**, **J**, and **L** HEYA8 cells were incubated in a control or cysteine-deprived medium with or without 100 μM NSC15364 or 400 μM DIDS for 16 h. **E**, **F** A representative flow cytometry histogram of C11-BODIPY staining (upper panels) and the average MFI ± SD of C11-BODIPY staining (low panels) (*n* = 3; **p < 0.01, ***p < 0.001, ns not significantly different). **G**, **H** A representative flow cytometry histogram of MitoPeDPP staining (upper panels) and the average MFI ± SD of MitoPeDPP staining (low panels) (*n* = 3; *p < 0.05, ***p < 0.001). **I**, **J** A representative flow cytometry histogram of MitoSOX. **K**, **L** A representative flow cytometry histogram of TMRE. Cys cysteine, MFI mean fluorescence intensity, NSC NSC15364, SD standard deviation, TMRE tetramethylrhodamine ethyl ester perchlorate.

We further investigated whether VDAC1 oligomerization is involved in cysteine deprivation-induced ferroptosis in other cancer cell types. Cysteine deprivation also induced ferroptosis in MDA-MB-231, a breast cancer cell line, and in HEYA8 cells, an ovarian cancer cell line (Fig. 8A–H). The VDAC1 oligomerization inhibitors DIDS and NSC15364 restored the cysteine deprivation-induced decrease in cell viability in a dose-dependent manner (Fig. 8A–D). Furthermore, DIDS reduced the increased lipid peroxidation and mitochondrial lipid peroxidation in cysteine-deprived cells (Fig. 8E–H). In cysteine-deprived MDA-MB-231 and HEYA8 cells, DIDS treatment decreased the elevated accumulation of mitochondrial ROS and MMP hyperpolarization (Fig. 8I–L). These results suggest that inhibition of VDAC1 oligomerization reduces cysteine deprivation-induced ferroptosis in various cancer cell lines.

## Discussion

Ferroptosis is an iron-dependent non-apoptotic form of cell death characterized by the accumulation of intracellular lipid reactive oxygen species (ROS) [2, 6]. The primary driving force of ferroptosis is cellular ROS stress generated by metabolic activities [36]. Excessive cellular ROS react with polyunsaturated fatty acids in the cell membrane, catalyzed by iron, initiating and propagating lipid peroxidation, which eventually leads to cell death [36]. Remarkably, cancer cells typically have a higher metabolic rate than normal cells, resulting in elevated ROS levels, making them inherently vulnerable to ferroptosis [37, 38]. The ferroptosis-inducing approach has been proven to be effective in overcoming resistance to standard-of-care therapies, including various targeted therapies, chemotherapy, and radiotherapy [12–15]. Here, we demonstrate that the metabolic activity of mitochondria plays a critical role in ferroptosis induced by cysteine deprivation. In particular, mitochondrial ROS and voltage-dependent anion channel-1 (VDAC1) oligomerization are essential for cysteine deprivation-induced ferroptosis.

Mitochondria serve as metabolic hubs and crucial sources of ROS [39]. The accumulation of ROS in the mitochondria has been reported to induce ferroptosis, and this process is inhibited by antioxidants targeting the mitochondria [24]. In this study, the mitochondria-targeting antioxidants MitoQ and MitoT blocked ferroptosis induced by cysteine deprivation, providing strong evidence linking mitochondrial ROS to cysteine deprivation-induced ferroptosis (Fig. 2). Most ROS are generated by the mitochondrial electron transport chain (ETC) during electron transport [40]. Treatment with inhibitors targeting mitochondrial complex I (Rotenone), complex II (DBM), complex III (Antimycin A), and complex IV (NaN_3_) for 12 h suppressed cysteine deprivation-induced ferroptosis. In particular, Antimycin A maintained its inhibitory effect on ferroptosis, even after 24 h (Fig. 3U). These results suggest that mitochondrial ETC activity is required to generate sufficient lipid ROS to initiate ferroptosis via cysteine deprivation. In a previous study, it was reported that mitochondrial membrane potential (MMP) and oxygen consumption increased during ferroptosis induced by cysteine deprivation [41]. Treatment with CCCP, a mitochondrial oxidative phosphorylation uncoupler, disrupted MMP and attenuated lipid ROS accumulation and ferroptosis under cysteine deprivation (Fig. 3B, C). These data suggest that MMP hyperpolarization during ferroptosis reflects increased mitochondrial ETC activity and mitochondrial respiration, leading to subsequent lipid ROS generation and accumulation.

VDAC1, a key regulator of mitochondrial function, is mainly expressed in the outer mitochondrial membrane (OMM) [27]. VDAC1 plays a critical role in promoting ferroptosis during tissue damage and organ dysfunction in various diseases including cancer, Alzheimer’s disease, liver injury, and myocardial infarction [31, 42–44]. Cysteine deprivation increased the formation of VDAC1 oligomers in cells overexpressing VDAC1 (Fig. 4A). Treatment with VDAC1 siRNA in these cells restored cell viability and alleviated the increased lipid peroxidation (Fig. 4G, H). Furthermore, the VDAC1 oligomerization inhibitors NSC15364 and DIDS reduced MMP hyperpolarization and alleviated elevated accumulation of mitochondrial ROS and increased oxygen consumption in response to cysteine deprivation or RSL3 (Figs. 5 and 7). These findings suggest that VDAC1 oligomerization induced by cysteine deprivation may contribute to ferroptosis. VDAC1 maintains mitochondrial calcium homeostasis by acting as a calcium ion channel [45]. VDAC1 oligomerization is associated with elevated ROS and Ca^2+^ levels [46]. However, in our study, the calcium chelator BAPTA did not inhibit cysteine deprivation-induced ferroptosis (Fig. 6). These results demonstrate that calcium ions are not associated with cysteine deprivation-induced ferroptosis.

In conclusion, cysteine deprivation- or RSL3-induced ferroptosis led to mitochondrial dysfunction, as evidenced by MMP hyperpolarization and increased oxygen consumption. Interestingly, the inhibition of VDAC1 oligomerization blocked ferroptosis by restoring mitochondrial dysfunction in cells deprived of cysteine or treated with RSL3. We highlighted that mitochondrial metabolic activity, which is regulated by VDAC1 oligomerization, plays an essential role in ferroptosis. This study provides insight into the role of VDAC1 oligomerization in the complex molecular network governing ferroptosis.

## Supplementary information

Raw data (WB)

## Data Availability

The data supporting the findings of this study are available from the corresponding author upon reasonable request.
