# Supplementary figures and images for: Inhibition of VDAC1 oligomerization blocks cysteine deprivation-induced ferroptosis via mitochondrial ROS suppression
Source: Cell Death Dis. 2024 Nov 9;15(11):811. doi: 10.1038/s41419-024-07216-1 (PMC11550314; doi:10.1038/s41419-024-07216-1)

Fig. 4A

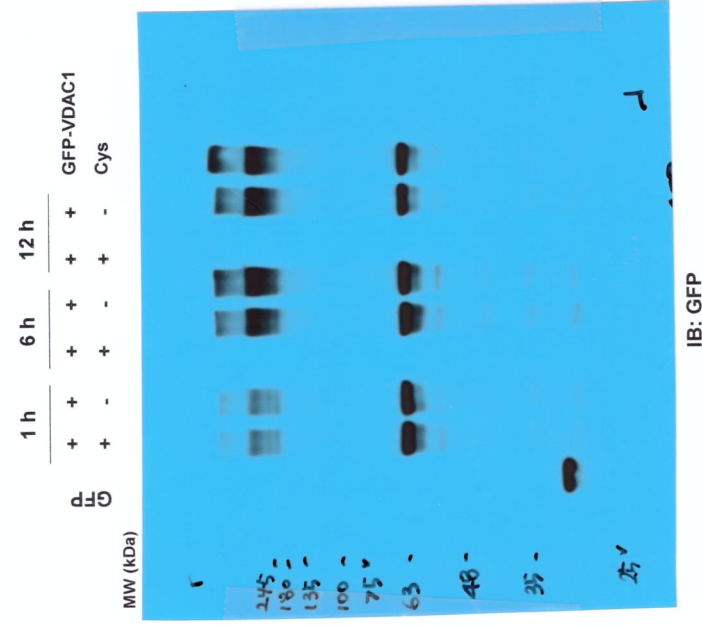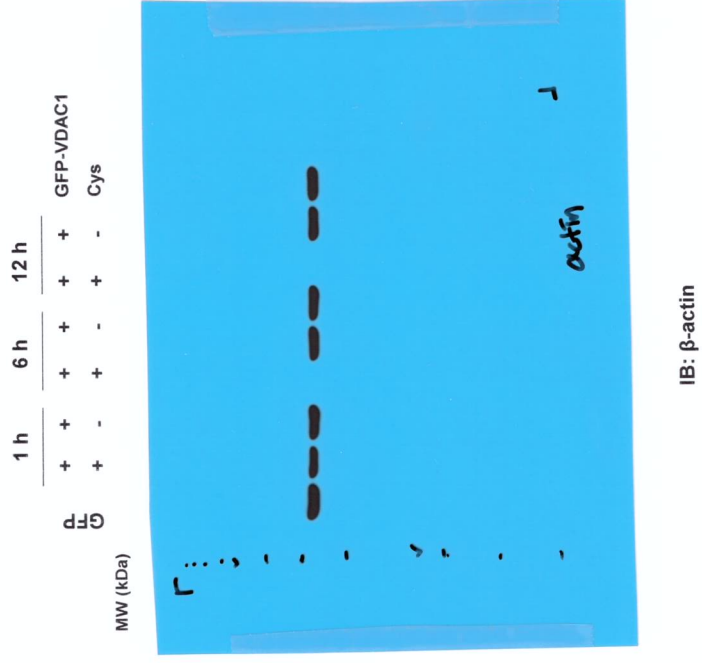

Fig. 4B

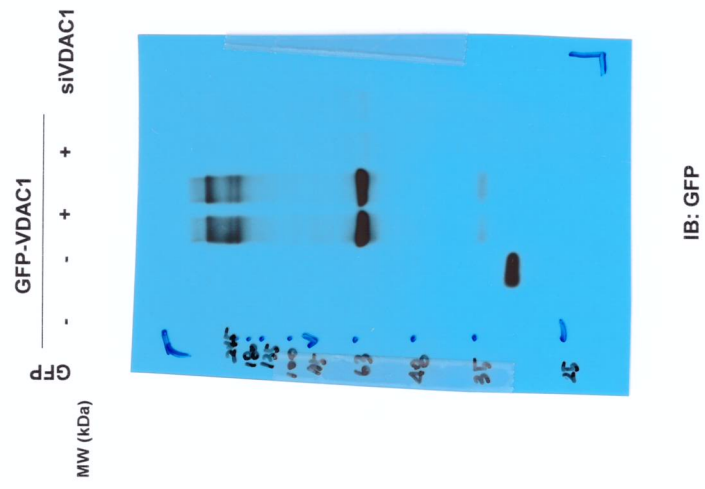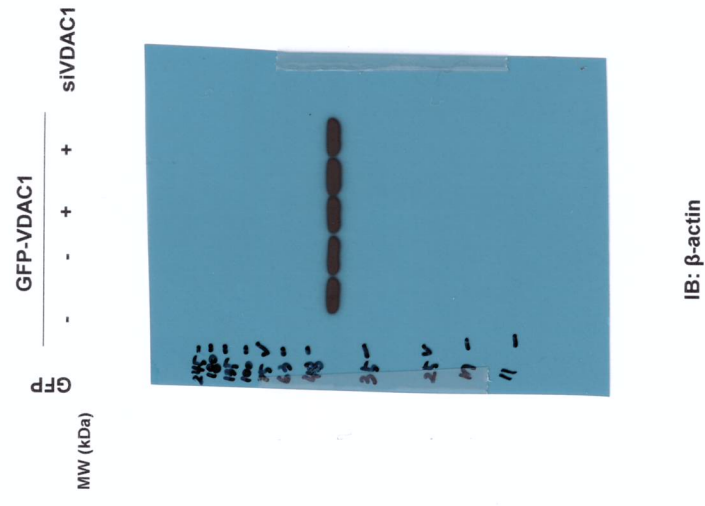

Fig. 4C

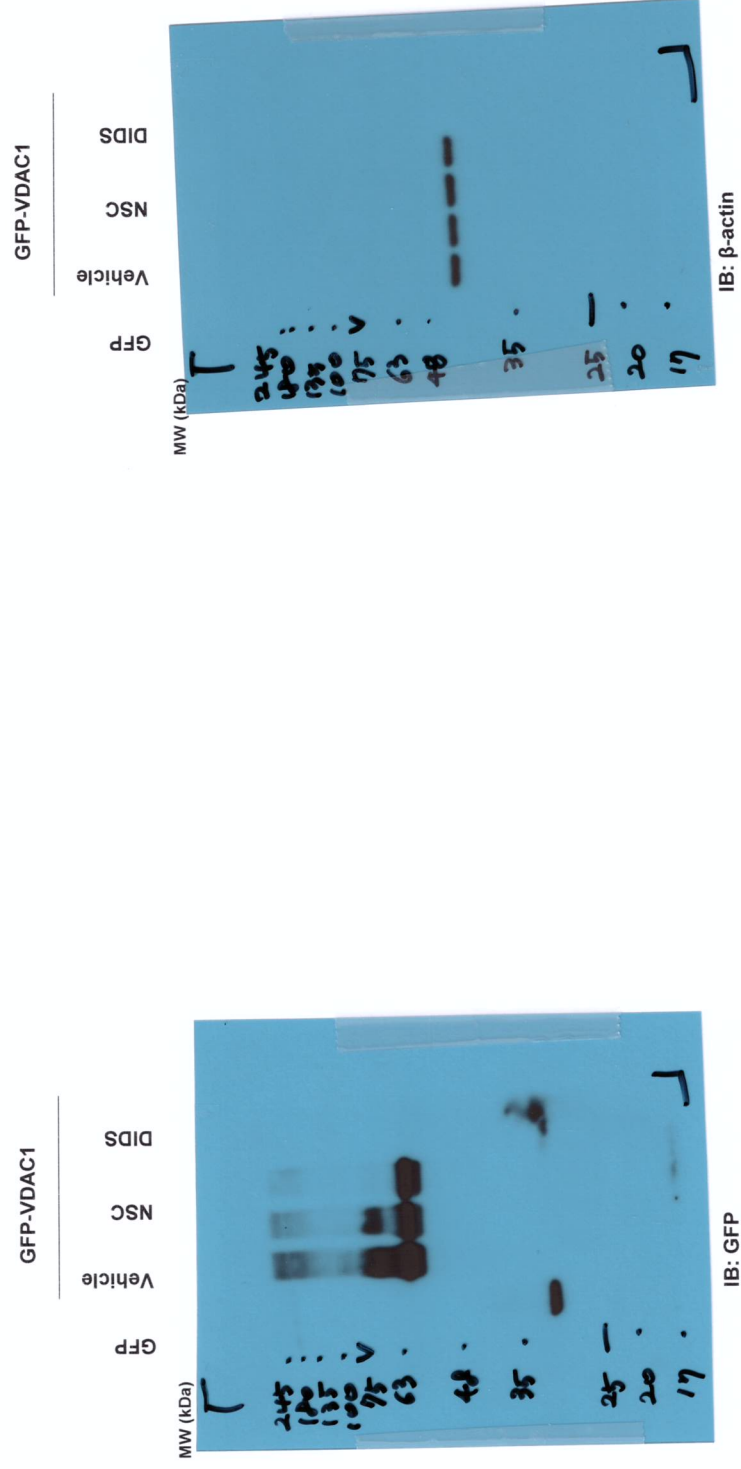

Supplement: Supplementary file 1 — Raw data (WB) [file 41419_2024_7216_MOESM1_ESM.pdf]
